# 

**Published:** 1871-04

**Authors:** 


### INDEX.

Abortion, Criminal .............. .... 621
Abstracts from German Journals.......
553, 585, 634
Academy of Medicine, Atlanta.
Organization of..................... 47
Proceedings of—19, 79, 128, 151, 216, 221
320, 330, 357, 387, 392, 401, 463, 526, 569
622, 660, 683.
Prosecution of.......................... 128
Academy of Medicine, Detroit.......... 154
Academy of Medicine, Meigs and Mason 260
Æsculapian Society Report on Surgery 340
Albuminuria, Clinical Lecture on...... 193
Albuminuria in Puerperal Convulsions
(Atlanta Academy of Medicine).. 320
Amnion, Dropsy of, with Triplets...... 199
Anæmia, with Epistaxis (Atlanta Academy of Medicine)................. 80
A New Departure....................... 291
Anus, Tissue of........................ 606
Atlanta Medical College...............50, 108
Alumni Meeting........................ 191
Clinic of............................... 82
Failure of its Enemies................ 180
Medical Clinic of................177, 213
Notice of.............................. 293
Preparatory Course.................. 732
Statement of Facts Concerning.... 109
Surgical Clinic of..................... 209
Atlanta Medical and Surgical Journal.. 45
Arsenic in Dyspepsia.................. 616
Arsenious Acid in Skin Diseases....... 256
Asylum, Inebriate...................... 662
Belladonna in Orchitis................. 475
In Incontinence of Urine.......... 56
Bibliographical.......................549, 725
Bismuth Subnitrate in Diarrhœa....... 62
Bladder, Inversion of.................. 606
Blood-Letting (Atlanta Academy Medicine)................................ 529
Brain, Wound of........................ 451
Injuries to Base of.................. 92
Buboes, Rapid Cure of.................. 58
Burns, Treatment of (Atlanta Academy of Medicine)......................... 79
Calhoun, Dr. A. W...................... 724
Cancer, Cundurango in.................. 240
Carbuncle, My Experience with a....... 558
Carbonic Acid Gas in Uterine Affections
(Atlanta Academy of Medicine).. 628
Cataract, Extraction in................ 405
Senile and Tramatic............... 611
Treated by Cups.................... 609
Cerebral Hemorrhage, Pathology of.... 43
Cerebro-Spinal Meningitis...........51, 709
Discussion of (Atlanta Academy of Medicine)..................21, 151, 696
Epidemica............................. 444
Successfully Treated................ 336
Childhood, Diseases Nervous System in 280
Chloral Hydrate, External Application 458
Dangerous Results from..............171
Death From............................. 162
In Incontinence of Urine.......... 61
Injurious Effects of (Atlanta Academy of Medicine).................. 81
In Singultus........................... 101
Overdose of............................. 163
Physiological Action of............. 652
Chloral Hydrate and Strychnine........ 61
Chorea, Notes on....................... 98
Clinical Lectures on Medicine.......... 32
Cloth Tents (Atlanta Academy of Medicine).........................51, 388, 401
Cloth Tents, Uterine................... 431
Colon, Puncture of..................... 598
Communication from Dr. J. J. Caldwell 148
Congenital Apnœa (Atlanta Academy of Medicine)............................ 387
Congestive Chill, Hæmaturia in........ 619
Correspondence, New York.............. 357
Editorial..............................420
Foreign................................ 545
Constipation, Obstinate (Atlanta Academy of Medicine).................. 463
Contraction of Muscles of Face, with Operation............................ 224
Coxalgia (Atlanta Academy of Medicine) 80
Cundurango a Failure.................. 548
Dactylitis Syphilitica.................. 299
Death, from Wound by a Pin (Atlanta Academy of Medicine).............. 218
From Hypodermic use of Morphine 546
Of Dr. O'Keefe...................... 61
Of Dr. Ridley........................ 666
Delivery, Premature.................... 146

Delirium Dremens, Digitalis in........ 57
Discussion of (Atlanta Academy of Medicine)........................ 324
Mode of Using Opium in............ 608
Diagnosis, of Syphilis................... 667
Of Phantom Abdominal Tumors... 670
Diphtheria (Atlanta Academy Medicine) 683
Diseases of the Genito-Uurinary Organs, New Instrument in ............. 506
Dislocation and Fracture of Femur... 298
Dressings, Surgical........................ 277
Dropsy, Acute Renal.......... .. ...... 90
Dysentery (Atlanta Academy Medicine) 693
Education, Medical........................ 600
Effects of White Sulphur Water, Virginia, in Inebriation.............. 711
Elbow Luxation Backward ........... 28
Electricity as a Therapeutic Agent (Atlanta Academy Medicine) .... 691
Electrolysis .... ........................ 229
Electrotherapy, Case of,............... .... 607
Empyema, New Mode of Drawing off Pus in (Atlanta Academy Medicine) 622
Endo-Metritis (Atlanta Academy Medicine).... ................ .. .... 222
Epileptic Convulsions (Atlanta Academy of Medicine) ...... ....... 693
Ergotine as a Hæmostatic............... 610
Eye, Extraction of........................ 551
Females, Diseases Peculiar to.......... 1
Fever, of Unusual Type (Atlanta Academy Medicine)...................... 324
Typhoid (Atlanta Academy of Medicine)............................ 398
Fevers, Low Continued.................. 303
Fistulæ, Hepatic......................... 88
Gestation, Hygiene of..........512, 569, 575
Georgia Lunatic Asylum.......... .... 723
Georgia Medical Association........... 721
Abstract of Proceedings........585, 634
Annual Meeting of............. .. . 732
Extract from Minutes of... ....... 724
Glandular Tumor, Cervical............... 17
Hæmaturia, Malarial (Atlanta Academy of Medicine)...................... 630
Hæmoptysis (Atlanta Academy of Medicine)................................ 216
Heart-Palpitation ... .................... 164
Hemorrhage, Post-Partem.... .. ...... 106
Ipecac in...... ...................... 285
Hernia, Aspirations in.................. 609
Reduced by Cold Water............ 62
Strangulated ........ .............. 338
Homœopathy.............................. 669
Hospitals, Mirror of.... ...... ........ 282
Hydrophobia.............. ............. 466
Hypodermic Medication.................. 129
Improvements in Medicine, Resume of 313
Incontinence of Urine.................... 56
Indian Springs, Properties of Water... 333
Inhalations in Pectoral Diseases........ 6
Infants and Children, Wasting Diseases 183
Injections, Sub-Cutaneous............... 256
Injury of Foot (Atlanta Academy Medicine)................................ 221
Insurance Incident........................ 59
Laminaria Tents, Notes on.............. 94
Laryngoscope, Management of the..... 368
Leucorrhœa in Children (Atlanta Academy of Medicine).... . ........ 465
Lithia . ........................ .. ...... 272
Macon Convention, Proceedings of..... 186
Magnesia Liq .............................. 657
Manilla Paper for Splints............... 22
Meddlesome Midwifery.................. 473
Medical Profession and Press ........ 659
Medical Profession in Georgia.......... 544
Middle Georgia Medical Society....664, 730
Menorrhagia, Carbolic Acid in (Atlanta Academy Medicine)................ 330
Menstruation and Lactation (Atlanta Academy of Medicine)............ 694
Morbus Brightii (Atlanta Academy Medicine)................................ 79
Nævoid Growths............................ 281
Nasal Spritz (Atlanta Academy of Medicine).... ............................ 81
New Apparatus for.... ............ 10
Nausea in Pregnancy..................... 624
New Arrangement......................... 417
Notes on New Remedies................. 204
Olla-Podrida, Professional............... 705
Onanism and Kindred Sins............. 644
Operations by Students.................. 290
Ophthalmia Neonatorum................ 654
Opium-Eating (Atlanta Academy Medicine).................... ..... 690
Orchitis (Atlanta Academy Medicine).. 80
Orthopœdic Apparatus................... 300
Ovarian Tumor (Atlanta Academy Medicine).................................. 397
Pamphlet of Dr. Crawford.............. 184
Paracentesis-Thoracis.. ............ .... 429
Pelvic Contraction, Delivery of Head in 283
Pepsin and Pancreatine, Solutlon of (Atlanta Academy Medicine)........ 38
Pessary, Meigs' Ring (Atlanta Academy Medicine........ ................. 392
Phlegmasia Doleus (Atlanta Academy of Medicine).... ...................... 152
Pinus Canadensis......................... 255
Placenta, Management of............... 288
Placenta Prævia, Digital Compression in 610
Pneumonia, Tubercular.................. 102
Poisoning from the Gases from the Explosion of Gunpowder........... 65
Presentation, Compound.... .......... 263
Progress........................ .......... 356
Prospects for the New Year............. 599
Pruritus Vulvæ, Treatment of........... 658

Puncture in Inflamed Testicle.......... 58
Publications Received................ 481
Puerperal Convulsions, Albuminuria in (Atlanta Academy Medicine)..... 320
Quackery in Medical Journalism....... 724
Quinine, Poisoning by................ 244
Recti-Linear Ecraseur, Artery Forceps. 383
Rectum, Injury of.................... 202
Reflections on Uterine Disease, as Connected with Phthisis Pulmonalis 677
Retention of Urine, Ice in............ 350
Review.......................410, 602
Reviews.............................. 460
Rheumatism, Acute.................... 70
Rush Medical College, Chicago......485, 731
Scarlatina, Peculiar Case of.......... 62
Scarlet Fever (Atlanta Academy Medicine)........................... 626
Spleen, Extraordinary Case of Enlargement of........................... 41
Nature of ........................ 59
Rupture of........................ 75
Small-Pox, Fatality of............... 60
Action of Light on............... 85
Small-Pox and Vaccination............ 55
Soap Made of Castor Oil.............. 176
Splints, Manilla Paper............... 52
Step in the Right Direction.......... 663
Strabismus, Convergent............... 563
Stricture of Urethra................. 260
Subscribers.......................... 54
Sun-Pains (Atlanta Academy Medicine) 659
Suppression of Menstrual Flow (Atlanta Academy of Medicine)........... 331
Supra-Pubic Puncture of the Bladder.. 714
Suspended Fœtal Animation........616, 710
Syphilis-Corpuscles.................. 718
Syphilis of Nervous System........... 271
Tænia in an Infant Five Days Old...... 610
Temperature During Pregnancy........ 268
Tetanus, Hydrate of Chloral in........ 286
Tongue, Excision of.................. 250
Tonsilotomy.......................... 62
Triplets, with Dropsy of the Amnion.. 199
Tumor, Abdominal (Atlanta Academy of Medicine)....................... 224
Fibrous of the Uterus............. 248
Typhlitis (Atlanta Academy Medicine). 225
Ulcer of Stomach, Perforating ........ 269
Umbilical Cord, Short (Atlanta Academy of Medicine)................ 79
Urethra, Stricture of................. 257
Uterine Compression, etc ............. 273
Uterine Diseases (Atlanta Academy of Medicine)........................ 19
Uterus, Probing of (Atlanta Academy of Medicine)...................... 526
Vaccination, Insufficiency of......... 671
Vaccine Crusts, Preservation of....... 40
Vaginismus........................... 412
Version, Cases of..................... 174

### PROSPECTUS OF THE ATLANTA MEDICAL AND SURGICAL JOURNAL.

---

The third series of this Journal is commenced with the view of making it a medium of communication between members of the Medical Profession, by which the advancement of science may be promoted, new practical truths disseminated, and a fraternal feeling and unity of purpose secured among honorable practitioners.

Its course, as heretofore, will be independent, yet, courteous and respectful in opposing error.

Medical journalism is more a "labor of love" than of pecuniary profit, and we hope practicing physicians in this and other States, will feel sufficient interest in the regular publication of such a work, to sustain it by cash subscriptions.

The Journal contains sixty-four pages, and will be published monthly at $3 a year, in advance.

The Editors propose, for this small sum, to afford physicians the current medical news. To do this, thirty or forty journals are thoroughly scanned monthly, and the cream of these publieations culled for our pages.

No personal feuds, parties or cliques, will receive encouragement at our hands. The advancement of medicine, as a science, the success and usefulness of the profession, and the highest standing of ethics and education will be advocated fearlessly on all occasions.

On business of any kind connected with the Journal, address "Editors ATLANTA MEDICAL AND SURGICAL JOURNAL."

Atlanta, Ga., April, 1871.

